# Social Distancing, Physical Activity, and COVID-19: Implications for Type 1 Diabetes Mellitus in Brazil

**DOI:** 10.3390/ijerph182312819

**Published:** 2021-12-05

**Authors:** Paulo H. C. de Vasconcelos, Daniela L. Gomes, Gabriela C. Uliana, Anselmo de A. Costa e Silva

**Affiliations:** 1 Nucleus of Behavior Theory Research, Federal University of Pará, Belém 66075-110, Brazil; pauloefpesquisa@gmail.com (P.H.C.d.V.); danielagomes@ufpa.br (D.L.G.); 2 Graduate Program in Physical Education, Federal University of Pará, Belém 66075-110, Brazil; anselmocs@ufpa.br

**Keywords:** social isolation, coronavirus infections, physical exercise

## Abstract

A lack of glycemic control and diabetes are risk factors for complications related to COVID-19, and social isolation can hinder adherence to physical activity. Thus, this study sought to assess the impacts of social distancing on the practice of physical activity of individuals with type 1 diabetes mellitus (T1DM). This was a transversal study carried out using an online form to collect sociodemographic, practice of physical activity, and social distancing data. Of the 472 participants, 85.6% reported that they were respecting the steps of social distancing. Social distancing affected the practice of physical activity in adherence to the habit of practicing in frequency, duration, and perception of change in intensity. An association was found between noticing a lot of stress in the home environment and stopping physical activity; lower levels of tolerance to social distancing were associated with less physical activity, and maintaining the habit of practicing physical activity was associated with decreasing the intensity of the practice. Hence, social distancing harmed the practice of physical activity as part of the treatment of individuals with T1DM, both in the habit of practicing and in the characteristics of these practices of physical activity, such as frequency, duration, and intensity.

## 1. Introduction

In December 2019, there began a global effort to tackle an epidemic that was first identified in the city of Wuhan, China, and later officially declared as a pandemic by the World Health Organization in March 2020. The disease, initially of unknown etiology, was later named coronavirus (COVID-19) and the causative virus SARS-CoV-2, with the first case reported in Brazil in late February 2020 [1,2,3]. A study showed that large cities displayed higher growth rates in the number of cases during the initial spread of COVID-19. However, these growth rates tended to decrease in large cities and to increase in small ones in the long-term course of the pandemic [4]. If death rates were equal to 2019 all-cause rates in the absence of COVID-19, COVID-19 death rates in 2021 would have already reduced *e*_0_ in 2021 by 1.8 years, which is larger than the reduction estimated for 2020 [5].

With a high prevalence in individuals with diabetes, the most common initial symptoms in this population are fever, cough, shortness of breath, loss of smell/taste, and headaches. Other less common symptoms are gastrointestinal symptoms, such as diarrhea and nausea, which can be observed right at the beginning of the infectious condition [6,7]. In the most critical cases, SARS-CoV-2 causes severe infection in the lower respiratory tract, which can lead to death [8].

Among the factors that increase the severity of COVID-19 infection, chronic diseases, such as obesity, diabetes mellitus (DM), and systemic arterial hypertension, in addition to advanced age are associated with higher mortality, hospitalizations, and worsening of respiratory conditions as a result of the infection [1,9,10,11]. Specifically in DM, patients with systemic acute respiratory syndrome (SARS) present a greater lack of glycemic control, leading to a greater risk of severe hypoxia and death, especially when the lack of control occurs during the course of the disease [11]. For individuals with type 1 diabetes mellitus (T1DM) and type 2 diabetes mellitus (T2DM), glycemic control is more complicated in patients with COVID-19 infection [12]. The difficulty in controlling blood glucose may originate from the characteristic of the action of the virus, which uses angiotensin-converting enzyme (ACE-2) receptors, which have higher expression in individuals with DM, to attack the islets of Langerhans. Initially, the virus attacks nasal, bronchial, and pneumocyte epithelial cells using ACE-2, which mediates the entry of coronavirus into host cells [8]. This explains, in part, the cases of critically ill patients needing higher doses of insulin, the cases of severe ketoacidosis on admission to hospitals, and the cases of acute diabetes in non-diabetic individuals [13,14,15]. From another perspective not linked to physiopathology, the anxiety generated by the stress of hospitalization, the difficulty in exercising, and the infectious situation itself as a whole affect glycemic control [12].

The management of DM is divided into four main pillars: insulin therapy, self-monitoring of blood glucose, physical activity, and adequate nutrition [16]. The negative impact on these four pillars has already been observed as a result of confinement and social isolation in Brazil, with an increase in unhealthy behavior related to diabetes: worsening in the frequency of self-monitoring of blood glucose, difficulty in obtaining treatment supplies, postponement of routine appointments, worsening in the quality of food, and especially worsening in physical activity habits [17].

Physical activity brings benefits to individuals who incorporate this habit into their routine and is considered one of the main aspects in the treatment of DM. Controlling blood glucose is the main benefit for this population, increasing insulin sensitivity, improving glycated hemoglobin (HbA1c), and decreasing insulin doses, which can generate effects up to 72 h after physical activity [18,19,20,21]. Although the benefits of glycemic control in T1DM are not as clear as in T2DM, the general benefits, the decrease in hypoglycemia and hyperglycemia episodes, reinforce the importance of physical activity. The decrease in blood pressure, the decrease in risk factors associated with DM (such as cardiovascular risk), the improvement in mental well-being, the increase in life expectancy, the increase in lean mass, and the improvement in body composition are other incentives for those who seek a better quality of life [19,21]. The literature also provides evidence of improvement in triglycerides, LDL, and waist circumference and shows that high volumes of aerobic training at moderate-to-vigorous intensity are associated with lower mortality and symptoms of anxiety/depression in both T1DM and T2DM [22,23].

The recommendations for this population are at least 150 min per week of physical activity with moderate-to-vigorous intensity or 75 min per week of intense physical activity in interval training. Within this minimum recommended practice time, two to three days a week of resistance training for the main muscle groups is also recommended, even in the scenario of the pandemic and social distancing, provided that all safety measures are taken respecting biosafety protocols. The total movement time must be within the recommended practice time, whatever the type of exercise [18,21,22,23]. For children and adolescents, the recommendation is at least 60 min a day of moderate-to-vigorous physical activity and three weekly sessions of intense physical activities that require muscle strength [22,23].

Recently, a systematic review with 66 studies, totaling 86,981 participants (13 to 86 years old) around the world, evaluated changes in the physical activity and sedentary behavior of individuals during the steps of social distancing, comparing physical activity profiles before and during isolation. Out of the analyzed studies involving only adults (45 studies), more than 50% (25 studies) of them reported a decrease in the time of physical activity. The rest, which evaluated changes not only in time, observed maintenance or a decrease in both the volume and intensity of physical activity. In the studies with children and adolescents (six studies), all observed a decrease in physical activity levels for this age group. The review also included studies with populations with special medical conditions, such as T1DM and T2DM and metabolic diseases. All found a decrease in physical activity time [24].

In a context in which physical inactivity can negatively impact the complications associated with DM and increase the morbidity and mortality of this population, the maintenance/adherence to the practice of physical activity is essential, especially in T1DM, regardless of the difficulties posed by social distancing [17,25]. Thus, we sought to investigate the impacts of social distancing on the practice of physical activity of individuals with T1DM.

## 2. Materials and Methods

### 2.1. Design and Study Participants

This was a transversal, descriptive, and analytical study, which was carried out in July 2020, a period in which most cities had already experienced social distancing in Brazil due to the COVID-19 pandemic [26,27].

Simple random sampling was performed with patients diagnosed with T1DM. The research was disseminated through social networks (Whatsapp^®^, Facebook^®^, and Instagram^®^) of an Extension and Research Project linked to a public university in northern Brazil. Inclusion criteria were age equal to or greater than 18 years of both genders and having a diagnosis of T1DM. As exclusion criteria, participants who marked any alternative other than the inclusion criteria were excluded, such as type 2 diabetes mellitus (T2DM), gestational diabetes, and other specific types; those who were unable to inform the type of diabetes they had; individuals who had answered the survey by third parties; and minors (−18 years old). These questions were asked at the beginning of the online questionnaire (better described later), also excluding people who did not complete the survey or did not agree with the informed consent form (ICF), option “I do not accept to participate in the survey”, available at the beginning of the questionnaire, below the link to the ICF. The collection began after consideration by the Ethics Committee of the Tropical Medicine Center of the Federal University of Pará (opinion 4.147.663).

### 2.2. Instrument

The survey was conducted using an online form with 30 objective questions and 4 simple subjective questions in the opinion survey format, according to Resolution No. 510/2016 [28], also complying with the legal requirements of Resolutions No. 466/2012 [29], which considers the Declaration of Helsinki for studies involving human beings. Participation was voluntary, not requiring any type of information in which it is possible to identify the participants. The ICF was made available in full on a link on the online platform before any question in the questionnaire. The form was built on the Google Forms^®^ platform and disseminated via the internet.

After receiving the invitation to participate in the study, through the aforementioned dissemination strategies, the participant was given a link to access the online questionnaire. The questionnaire consisted of four axes, namely:

(a) Sociodemographic: containing questions regarding biological sex, age, place of residence (state, city, neighborhood), housing condition (presence of open area in the residence and perception of comfort), level of education, number of people living in the same household, and number of people with diabetes in the same household.
(b) Financial status: family income, employment status, emergency aid, and donations.
(c) Physical activity: the practice of physical activity before and during social isolation (which activity, how many times a week, how long, domestic activity).
(d) Social isolation: type of isolation, opinion, impact of isolation, expectation, stress in the home environment, and changes in sleep.

The ICF and the questionnaire can be accessed by the link https://docs.google.com/forms/d/1FcflkKrbg_74jOH2n6Yd1S_Xdt48kzzvq8sImWb0APM/edit (accessed on 31 July 2020).

### 2.3. Data Analysis

For statistical analysis, the Statistical Package for Social Sciences (SPSS) software, version 21, was used. Descriptive results were expressed in absolute frequency and proportion. Initially, the simple chi-square test was applied to verify if the sample distribution was homogeneous among the categories in the descriptive stage regarding sociodemographic, physical activity, and social isolation data. In the analytical step, first, a variable, “Change in the practice of physical activity”, was created, where we unified the data “Practice of physical activity before isolation” and “Practice of physical activity during isolation” so that, for statistical purposes, we could have information about the difference in adherence before and during social isolation in one single variable. Thus, the individuals who answered whether they practiced physical activity before (yes or no) and if they practiced physical activity during (yes or no) could be classified as “Remained active” (yes and yes), “Remained inactive” (no and no), “Started the practice” (no and yes), and “Stopped the practice” (yes and no). For a better example, if a participant answered that they practiced physical activity before isolation and also continued to practice during isolation, they were classified as “remained active”, while a participant who answered that they did not practice physical activity before isolation but started during isolation was classified as “Beginning the practice”. This line was followed for the four categories mentioned above. With this new variable in hand, the chi-square of independence and Pearson correlation test were applied, considering the sample “n” and the sample distribution, respectively. Chi-square was used to test the associations between “Change in the practice of physical activity” and sociodemographic, physical activity, and social isolation variables. A level of statistical significance of *p* < 0.05 was considered.

## 3. Results

The survey was answered by 576 people, of which 472 met the study criteria (T1DM adults) and most of whom were female and aged between 25 and 44 years. The sociodemographic information is better described in Table 1 with data in absolute numbers and percentages.

Among the participants, 48.7% practiced physical activity during the period of social distancing, which is less compared to the period before the pandemic when there was greater adherence to the practice. Data on physical activity are available in Table 2, classified as before and during social distancing, expressed in absolute numbers and percentages.

As for data on social distancing, most participants reported that they were respecting the measures of social distancing and were willing to stay socially distanced as long as necessary to face the pandemic. Information regarding the data collected on social distancing can be found in Table 3 and is also expressed in absolute numbers and percentages.

Investigating the possible relationships between the variables that could interfere with the change in the physical activity profile in the face of the pandemic scenario, being male was directly associated with maintaining the practice of physical activity (*p* = 0.036). Furthermore, having a graduate degree was associated with maintaining the practice of physical activity and having only a high school education was associated with maintaining physical inactivity (*p* = 0.017). No association was found between adherence to the steps of social distancing and change in the practice of physical activity. However, when testing the association between tolerance to reported social distancing and changes in the habit of physical activity (*p* = 0.045), it was observed that not being able to spend an entire month in isolation was associated with stopping physical activity, while being willing to stay in isolation for the time necessary to face the pandemic was inversely associated with stopping physical activity. Furthermore, claiming to be able to stay socially distanced for between one and two months was associated with maintaining the practice of physical activity (Table 4). This shows that good tolerance to the isolation scenario minimizes the negative impacts of social distancing on the habit of practicing physical activity. This finding gains more strength when we analyze the relationship between stress in the isolated home environment and changes in physical activity. Reporting little stress in the home environment was associated with maintaining the practice of physical activity, and also, reporting a lot of stress in the home environment was associated with stopping physical activity (*p* < 0.000) (Table 4).

When observing the relationship between intensity and change in the habit of practicing physical activity, it was found that maintaining the habit of practicing physical activity was associated with decreasing the intensity of the practice (*p* < 0.000). This suggests a tendency among individuals who remained active to decrease activity intensity. As for the group of 27.54% (*n* = 130) who stopped practicing physical activity, to which no change in intensity can be attributed, a negative effect of social distancing was observed both in the change in the habit of practicing and in the intensity (Table 4).

## 4. Discussion

To date, among the searches in the current literature on the subject, this was the first study in which associations between tolerance to social isolation, stress in the home environment, and changes in the practice of physical activity in individuals with T1DM were tested. As discussed below, such data indicate that good tolerance to isolation conditions can minimize the negative impacts of social distancing on the habit of practicing physical activity.

Most of the participants were female. Data from the Ministry of Health indicate that females, in general, express greater concerns and care about their health. Moreover, females may be more interested in health research [30].

In the present study, it was observed that the perception of a lot of stress in the home environment was associated with stopping physical activity. The impact of social distancing on people’s mental health could be observed in the systematic review by Xiong et al. [31], where there was a high prevalence of symptoms related to anxiety and depression in all nineteen selected studies, mainly in females, people under 40 years of age, and people with chronic diseases. A study of 399 individuals (56.4% female, 41.9% male, and 1.7% non-binary/trans) practicing social distancing due to the new coronavirus pandemic examined changes in behavior related to good health and concluded that there are negative changes in mental health (anxiety, depression, fatigue, confusion, tension, and anger), and these characteristics are associated with worse physical activity. Out of the 399 individuals, 24.8% (*n* = 99) reported doing much less physical activity compared to the previous period and 22.6% (*n* = 90) reported practicing a little less physical exercise. In addition, the mean scores on the tests related to negative mood changes were higher in the groups that practiced less physical activity compared to the pre-pandemic period [32]. This panorama, combined with the understanding that diabetes is associated with a higher probability of being diagnosed with anxiety disorder and with presenting symptoms of depression [33], reinforces the idea that a scenario of social distancing can increase stress, which in turn can generate a lack of glycemic control in individuals with T1DM. Combating stress involves an active lifestyle and self-care, which can be directly affected by the steps of social distancing [16].

The negative impact of social distancing on physical activity in individuals with T1DM was also observed in the study by Verma et al. [34], in which there was a worsening in glycemic control and a decrease in self-care habits. Out of 52 participants (30 female and 22 male), 36.5% decreased the practice of physical activity. In this same study, it was possible to observe a significant increase (*p* < 0.001) in mean blood glucose from 212.3 mg/dl to 276.9 mg/dl and glycated hemoglobin (HbA1c) of 8.8% (73 mmol/mol) to 10% (86 mmol/mol). Another study, with 150 participants (93 male and 53 female) with T2DM, looked at changes in lifestyle and practices related to diabetes treatment after 45 days in lockdown. About 42% reported a decrease in the duration of physical activity practice. There was also weight gain in 19% of them, 23% reported decreased blood glucose self-monitoring, and 87% reported increased mental stress [35].

The study by Assaloni et al. [36], carried out in Italy with 154 individuals (54.5% men) with T1DM, also observed a reduction in the practice of physical activity and an increase in blood glucose averages during the quarantine period. The average number of minutes of daily exercise, the average number of steps taken per day, and the questionnaire score related to the practice of physical activity were significantly lower compared to the pre- lockdown period (*p* < 0.001). Likewise, in the study by Pal et al. [37], carried out with 30 participants (87.5% female) with T1DM, it was found that 27 (90%) participants reduced the practice of physical activity in some way during the period of social distancing, and, among these, the habits of 10 (33%) individuals were interrupted in full. In addition, there was a worsening of glycemic control in the group in which physical activity was reduced or interrupted.

A Brazilian study with 1701 participants (75.5% female) sought to observe the impacts of social isolation on individuals with T1DM (60.7%), type 2 (30.7%), and other types of diabetes (8.6%). The authors observed that 59.5% of them reduced the practice of physical activity, 33.57% maintained the same pattern, and only 6.96% increased the practice. Within the category of those who reduced the practice of physical activity, 44.8% were classified as a great reduction and 14.7% as a modest reduction; however, the criteria for these classifications are not clear in the study [17]. The study by Capaldo et al. [38], with 207 adults (111 male and 96 female) with T1DM, observed a significant reduction (*p* > 0.001) in the reported practice of physical activity. Out of the 182 participants who reported practicing physical activity during social distancing, 127 (70%) reported a reduction in the habit, 30 (16%) reported keeping the same routine, and 25 (14%) reported an increase in the practice of physical activity.

In contrast, Yan et al. [39] found improvements in the habit of practicing physical activity in T1DM and T2DM individuals. Out of a total of 585 participants (60.3% male), 73.8% (*n* = 432) met the minimum recommendations of practicing at least 150 weekly minutes of physical activity with light-to-moderate intensity, and 67.7% (*n* = 396) increased the practice of physical activity both in frequency and duration compared to the period prior to social distancing (*p* < 0.001). The authors suggest that individuals with diabetes perceive themselves as at greater risk of infection by COVID-19 compared to non-diabetics and, therefore, are more concerned with taking care of their health so as not to have worse clinical outcomes if they are infected.

In general, the pandemic situation in which Brazil is inserted seems to affect habits related to physical activity in individuals with T1DM. Since longer training programs (>12 weeks) with higher frequencies (>3 sessions/week) and longer durations (>60 min/session) have been shown to be more efficient in improving glycemic control, the reduction in these elements due to social distancing makes it difficult for this population to adopt an active lifestyle [40]. Physical activity should be considered as an important method of prevention of viral diseases, and when exercise is performed in forested areas, these protective health benefits may be increased, helping to alleviate stress and anxiety, lowering cortisol levels, and, consequently, helping the proper functioning of the immune system [41].

Furthermore, it was observed that being male was associated with maintaining the practice of physical activity. This corroborates the data from Agra, Montenegro, and Santos (2020) [42], which indicated that in all Brazilian states and in the federal district, males tend to be more physically active than females, both in the habit of regular physical activity and in the practice of physical activity in addition to daily activities. The hypothesis that could explain this phenomenon is that being female often requires carrying out household chores, taking care of the children and the house, in addition to their work, which can lead to more neglect of physical activity, as it requires greater availability of reserved time for practice.

### Limitations

It is important to emphasize that this research has some limitations, such as the fact that it was carried out through an online platform, which can cause a reduction in the population studied, excluding those who do not have access to such means. Another aspect was the non-assessment of the participants’ glycemic control, preventing a more reliable interpretation of the impacts of distancing on the adherence to the practice of physical activity and on the glycemic control of individuals with T1DM. It is suggested that future studies seek a more homogeneous distribution of the sample within all Brazilian regions and evaluate the glycemic control of the participants in order to test the possible associations between adherence to physical activity in social distancing scenarios and impacts on disease management.

## 5. Conclusions

According to the aspects presented, it is concluded that social distancing and some of its consequences have harmed the practice of physical activity as part of the treatment of individuals with T1DM, affecting the habit of practicing and the characteristics of these practices of physical activity, such as frequency, duration, and intensity. Therefore, it is possible to suggest structured digital programs of encouragement and guidance regarding the practice of physical activity to help this population in the scenario of social distancing, with relevance also in the post-pandemic period when the professionals responsible for the prescription and guidance of exercises can adapt their practices, incorporating digital means for situations that require remote assistance, helping to improve life expectancy.

## Figures and Tables

**Table 1:** Sociodemographic characterization of individuals with type 1 diabetes during the COVID-19 pandemic in Brazil.

	*n* (%)	*p*-Value *
Sex		
Female	406 (86.0)	<0.000 ^†^
Male	66 (14.0)	
Age (years old)		
18–24	161(34.1)	<0.000 ^†^
25–44	269 (57.0)	
≥45	42 (8.9)	
Macro-region		<0.000 ^†^
North	33 (7.0)	
Northeast	97 (20.6)	
Midwest	37 (7.8)	
Southeast	222 (47.0)	
South	83 (17.6)	
City		
State capital or metropolitan region	295 (62.5)	<0.000 ^†^
State inland	177 (37.5)	
District		<0.000 ^†^
Favela or community	10 (2.1)	
Periphery	79 (16.7)	
Middle class	260 (55.1)	
Upper class	73 (15.5)	
Rural area	20 (4.2)	
None of the alternatives	30(6.4)	
Perception of size of residence		<0.000 ^†^
Excellent	215 (45.6)	
Good	193 (40.9)	
Regular	57 (12.1)	
Bad	6 (1.3)	
Terrible	1 (0.2)	
Presence of open area in the residence		
Yes	339 (71.8)	<0.000 ^†^
No	133 (28.2)	
Schooling		<0.000 ^†^
Elementary School	4 (0.8)	
High School	21 (4.4)	
Technician	75 (15.9)	
Undergraduate	135 (28.6)	
Postgraduate	237 (50.2)	
Family income during social isolation		<0.000 ^†^
<1 MW	19 (4.0)	
≥1 and ≤2 MW	134 (28.4)	
≥3 and <5 MW	153 (32.4)	
≥5 and <10 MW	103 (21.8)	
≥10 and <20 MW	46 (9.7)	
≥20 MW	17 (3.6)	
Impact on income during social isolation		
Decreased by more than half the usual salary	94 (19.9)	<0.000 ^†^
Decreased less than half the usual salary	127 (26.9)	
Family income remained in the same range	239 (50.6)	
Family income increased	6 (1.3)	
There was no family income before the pandemic	6 (1.3)	
Impact on employment during social isolation		
Continued working normally	286 (60.6)	<0.000 ^†^
Became unemployed	71 (15.0)	
Was temporarily removed	96 (20.3)	
No one in the house was employed	19 (4.0)	
*Emergency Aid*		
Yes	185 (39.25)	<0.000 ^†^
No	287 (60.8)	

* Chi-squared. ^†^ Statistical significance; MW = minimum wage.

**Table 2:** Association between characteristics of social isolation and physical activity in individuals with type 1 diabetes during the COVID-19 pandemic in Brazil.

	Social Distancing			
	Before	*p*-Value *	During	*p*-Value *
	*n* (%)		*n* (%)	
Physical activity practice				
Yes	315 (66.7)	<0.000 ^†^	230 (48.7)	0.613
No	157 (33.3)		242 (51.3)	
Weekly frequency of physical activity				
Once a week	8 (1.7)	<0.000 ^†^	11 (2.3)	<0.000 ^†^
2 times a week	47 (10.0)		41 (8.7)	
3 times a week	80 (16.9)		57 (12.1)	
4 times a week	53 (11.2)		38 (8.1)	
5 times a week	85 (18.0)		51 (10.8)	
6 times a week	32 (6.8)		18 (3.8)	
Every day without a break	10 (2.1)		14 (3.0)	
Did not practice physical activity	157 (33.3)		242 (51.3)	
Average duration of physical activity				
<30 min	8 (1.7)	<0.000 ^†^	22 (4.7)	<0.000 ^†^
30 min	21 (4.4)		62 (13.1)	
>30 and ≤60 min	197 (41.7)		114 (24.2)	
>60 min	89 (18.9)		32 (6.8)	
Did not practice physical activity	157 (33.3)		242 (51.3)	
Perception of change in intensity of physical activity				
Maintained the intensity of before	#	#	37 (7.8)	<0.000 ^†^
Decreased intensity	#		121 (25.6)	
Increased intensity	#		48 (10.2)	
I couldn’t tell	#		24 (5.1)	
Did not practice physical activity	157 (33.3)		242 (51.3)	
Domestic activities				
Yes, every week	324 (68.6)	<0.000 ^†^	367 (77.8)	<0.000 ^†^
Yes, once a month	68 (14.4)		62 (13.1)	
No, never did	80 (16.9)		43 (9.1)	

* Chi-squared. ^†^ Statistical significance; # No change.

**Table 3:** Characterization of the social isolation of individuals with type 1 diabetes during the COVID-19 pandemic in Brazil.

	*n* (%)	*p*-Value *
Adherence to social distancing		
Respected social distance	404 (85.6)	<0.000 ^†^
Did not respect social distancing	68 (14,4)	
Tolerance to social distancing reported		
Can’t stay a whole month in this condition	56 (11.9)	<0.000 ^†^
Can stay between one and two months	62 (13.1)	
Can stay longer than two months	24 (5.1)	
Are you willing to stay as long as necessary	330 (69.9)	
Perception of the impact of social distancing		
Social life	188 (39.8)	<0.000 ^†^
Financial	147 (31.1)	
Health	114 (24.2)	
Others	23 (4.9)	
Stress in the home environment reported		
Did not notice any stress	71 (15.0)	<0.000 ^†^
Realized little stress	238 (50.4)	
Realized a lot of stress	163 (34.5)	

* Chi-squared. ^†^ Statistical significance.

**Table 4:** Association between sociodemographic characteristics and social isolation with the practice of physical activity of individuals with type 1 diabetes during the COVID-19 pandemic in Brazil.

	Change in Physical Activity Practice				
	Remained Active	Remained Inactive	Started the Practice	Discontinued Practice	*p*-Value *
	*n* (%)	*n* (%)	*n* (%)	*n* (%)	
Gender					
Male	36 (7.6) +	10 (2.1)	4 (0.8)	16 (3.4)	0.036 ^†^
Female	148 (31.4)	103 (21.8)	41 (8.7)	114 (24.2)	
Schooling					
Elementary School	0 (0.0)	2 (0.4)	1 (0.2)	1 (0.2)	0.017 ^†^
High School	2 (0.4) −	11.(2.4) +	1 (0.2)	7 (1.5)	
Technician	21.(4.5)	16 (3.5)	10 (2.2)	18 (3.9)	
Undergraduate	48 (10.4)	30 (6.5)	13 (2.8)	44 (9.5)	
Postgraduate	106 (22.9) +	52 (11.3)	19 (4.1)	60 (13.0)	
Adherence to social distancing					
Yes	163 (34.5)	94 (19.9)	39 (8.3)	108 (22.9)	0.461
No	21 (4.4)	19 (4.0)	6 (1.3)	22 (4.7)	
Tolerance to social distancing reported					
Can’t stay a whole month in this condition	14 (3.0) −	13 (2.8)	5 (1.1)	24 (5.1) +	0.045 ^†^
Can stay between one and two months	32 (6.8) +	8 (1.7) −	5 (1.1)	17 (3.6)	
Can stay longer than two months	10 (2.1)	5 (1.1)	1 (0.2)	8 (1.7)	
Are you willing to stay as long as necessary	128 (27.1)	87 (18.4)	34 (7.2)	81 (17.2) −	
Perception of change in intensity of physical activity					
Maintained the intensity of before	30 (13.0)	#	7 (3.0)	#	<0.000 †
Decreased intensity	116 (50.4) +	#	5 (2.2) −	#	
Increased intensity	29 (12.6) −	#	19 (8.3) +	#	
I couldn’t tell	9 (3.9) −	#	14 (6.1) +	#	
Did not practice physical activity	0 (0)	#	0 (0)	#	
Perceived stress in the home environment					
Did not notice any stress	31 (6.6)	19 (4.0)	10 (2.1)	11 (2.3) −	0.001 ^†^
Realized little stress	107 (22.7) +	48 (10.2)	24 (5.1)	59 (12.5)	
Realized a lot of stress	46 (9.7) −	46 (9.7)	11 (2.3)	60 (12.7) +	

* Chi-squared. ^†^ Statistical significance; residue analysis: (+) significant association, (−) negative significant association.

## Data Availability

The data presented in this study are available on request from the corresponding author. The data are not publicly available because the research was conducted through an online form that allows access to other data not used in this article.

## References

[1] Deng S., Peng H. (2020). Characteristics of and public health responses to the coronavirus disease 2019 outbreak in China. J. Clin. Med..

[2] Lai C.C., Shih T.P., Ko W.C., Tang H.J., Hsueh P.R. (2020). Severe acute respiratory syndrome coronavirus 2 (SARS-CoV-2) and coronavirusdisease-2019 (COVID-19): The epidemic and the challenges. Int. J. Antimicrob. Agentes.

[3] Brasil Ministério da Saúde (2020). Guia de Vigilância Epidemiológica: Emergência de Saúde Pública de Importância Nacional Pela Doença Pelo Coronavírus 2019.

[4] Ribeiro H.V., Sunahara A.S., Sutton J., Perc M., Hanley Q.S. (2020). City size and the spreading of COVID-19 in Brazil. PLoS ONE.

[5] Castro M.C., Gurzenda S., Turra C.M., Kim S., Andrasfay T., Goldman N. (2021). Reduction in life expectancy in Brazil after COVID-19. Nat. Med..

[6] Singh A.K., Gupta R., Ghosh A., Misra A. (2020). Diabetes in COVID-19: Prevalence, pathophysiology, prognosis and practical considerations. Diabetes Metab. Syndr..

[7] Hussain A., Bhowmik B., do Vale Moreira N.C. (2020). COVID-19 and diabetes: Knowledge in progress. Diabetes Res. Clin. Pract..

[8] Wiersinga W.J., Rhodes A., Cheng A.C., Peacock S.J., Prescott H.C. (2020). Pathophysiology, transmission, diagnosis, and treatment of coronavirus disease 2019 (COVID-19): A review. JAMA.

[9] Chen Y., Gong X., Wang L., Guo J. (2020). Effects of hypertension, diabetes and coronary heart disease on COVID-19 diseases severity: A systematic review and meta-analysis. medRxiv.

[10] Bornstein S.R., Rubino F., Khunti K., Mingrone G., Hopkins D., Birkenfeld A.L., Boehm B., Amiel S., Holt R.I., Skyler J.S. (2020). Practical recommendations for the management of diabetes in patients with COVID-19. Lancet Diabetes Endocrinol..

[11] Yang J.K., Feng Y., Yuan M.Y., Yuan S.Y., Fu H.J., Wu B.Y., Sun G.Z., Yang G.R., Zhang X.L., Wang L. (2006). Plasma glucose levels and diabetes are independent predictors for mortality and morbidity in patients with SARS. Diabetic. Med..

[12] Zhou J., Tan J. (2020). Diabetes patients with COVID-19 need better blood glucose management in Wuhan, China. Metab. Clin. Exp..

[13] Yang J.K., Lin S.S., Ji X.J., Guo L.M. (2010). Binding of SARS coronavirus to its receptor damages islets and causes acute diabetes. Acta Diabetol..

[14] Hoffmann M., Kleine-Weber H., Schroeder S., Krüger N., Herrler T., Erichsen S., Schiergens T.S., Herrler G., Wu N.H., Nitsche A. (2020). SARS-CoV-2 cell entry depends on ACE2 and TMPRSS2 and is blocked by a clinically proven protease inhibitor. Cell.

[15] Bindoma S.M., Lazartigues E. (2009). The sweeter side of ACE2: Physiological evidence for a role in diabetes. Mol. Cell. Endocrinol..

[16] Sociedade Brasileira de Diabetes (SBD) (2019). Diretrizes da Sociedade Brasileira de Diabetes (2019–2020).

[17] Barone M.T.U., Harnik S.B., de Luca P.V., Lima B.L.S., Wieselberg R.J.P., Ngongo B., Pedrosa H.C., Pimazoni-Netto A., Franco D.R., Marinho de Souza M.F. (2020). The impact of COVID-19 on people with diabetes in Brazil. Diabetes Res. Clin. Pract..

[18] Denay K.L., Breslow R.G., Turner M.N., Nieman D.C., Roberts W.O., Best T.M. (2020). ACSM call to action statement: COVID-19 considerations for sports and physical activity. Curr. Sports Med. Rep..

[19] McCarthy O., Moser O., Eckstein M.L., Deere R., Bain S.C., Pitt J., Bracken R.M. (2019). Resistance isn’t futile: The physiological basis of the health effects of resistance exercise in individuals with type 1 diabetes. Front. Endocrinol..

[20] Colberg S.R., Sigal R.J., Fernhall B., Regensteiner J.G., Blissmer B.J., Rubin R.R., Chasan-Taber L., Albright A.L., Braun B., American College of Sports Medicine (2010). Exercise and type 2 diabetes: The american college of sports medicine and the american diabetes association: Joint position statement executive summary. Diabetes Care.

[21] Church T.S., Blair S.N., Cocreham S., Johannsen N., Johnson W., Kramer K., Mikus C.R., Myers V., Nauta M., Rodarte R.Q. (2010). Effects of aerobic and resistance training on hemoglobin A1c levels in patients with type 2 diabetes: A randomized controlled trial. JAMA.

[22] American Diabetes Association (2019). Lifestyle management: Standards of medical care in diabetes—2019. Diabetes Care.

[23] American Diabetes Association (2021). Facilitating behavior change and well-being to improve health outcomes: Standards of medical care in diabetes—2021. Diabetes Care.

[24] Stockwell S., Trott M., Tully M., Shin J., Barnett Y., Butler L., McDermott D., Schuch F., Smith L. (2021). Changes in physical activity and sedentary behaviours from before to during the COVID-19 pandemic lockdown: A systematic review. BMJ Open Sp. Ex. Med..

[25] Roschel H., Artioli G.G., Gualano B. (2020). Risk of increased physical inactivity during COVID-19 outbreak in older people: A call for actions. J. Am. Geriatr. Soc..

[26] UOL. https://noticias.uol.com.br/saude/ultimas-noticias/redacao/2020/05/09/saiba-onde-ja-foi-decretado-o-lockdown-no-brasil.htm.

[27] Correio Braziliense. https://www.correiobraziliense.com.br/app/noticia/brasil/2020/05/08/interna-brasil,852582/lockdown-avanca-pelo-pais-e-chega-a-18-cidades-de-cinco-estados-veja.shtml.

[28] Brasil (2016). Resolução Nº510 de 07 de Abril de 2016, do Conselho Nacional de Saúde do Ministério da Saúde. Diário Oficial da União.

[29] Brasil (2012). Resolução Nº466 de 12 de Dezembro de 2012. Dispõe Sobre as Diretrizes e Normas Para Pesquisas Com Seres Humanos. Diário Oficial da União.

[30] Brasil (2011). Ministério da Saúde. Política Nacional de Atenção Integral à Saúde da Mulher: Princípios e Diretrizes.

[31] Xiong J., Lipsitz O., Nasri F., Lui L.M.W., Gill H., Phan L., Chen-Li D., Lacobucci M., Ho R., Majeed A. (2020). Impact of COVID-19 pandemic on mental health in the general population: A systematic review. J. Affect. Disord..

[32] Ingram J., Maciejewski G., Hand C.J. (2020). Changes in diet, sleep, and physical activity are associated with differences in negative mood during COVID-19 lockdown. Front. Psychol..

[33] Smith K.J., Béland M., Clyde M., Gariépy G., Pagé V., Badawi G., Rabasa-Lhoret R., Schmitz N. (2013). Association of diabetes with anxiety: A systematic review and meta-analysis. J. Psychosom. Res..

[34] Verma A., Rajput R., Verma S., Balania V.K.B., Jangra B. (2020). Impact of lockdown in COVID-19 on glycemic control in patients with type 1 Diabetes Mellitus. Diabetes Metab. Syndr..

[35] Ghosh A., Arora B., Gupta R., Anoop S., Misra A. (2020). Effects of nationwide lockdown during COVID-19 epidemic on lifestyle and other medical issues of patients with type 2 diabetes in north India. Diabetes Metab. Syndr..

[36] Assaloni R., Pellino V.C., Puci M.V. (2020). Coronavirus disease (COVID-19): How does the exercise practice in active people with type 1 diabetes change? A preliminary survey. Diabetes Res. Clin. Pract..

[37] Pal R., Yadav U., Verma A., Bhadada S.K. (2020). Awareness regarding COVID-19 and problems being faced by young adults with type 1 diabetes mellitus amid nationwide lockdown in India: A qualitative interview study. Prim. Care Diabetes.

[38] Capaldo B., Annuzzi G., Creanza A., Giglio C., De Angelis R., Lupoli R., Masulli M., Riccardi G., Rivellese A.A., Bozzetto L. (2020). Blood glucose control during lockdown for COVID-19: CGM metrics in Italian adults with type 1 diabetes. Diabetes Care.

[39] Yan A.F., Sun X., Zheng J., Mi B., Zuo H., Ruan G., Hussain A., Wang Y., Shi Z. (2020). Perceived risk, behavior changes and Health-related outcomes during COVID-19 pandemic: Findings among adults with and without diabetes in China. Diabetes Res. Clin. Pract..

[40] MacMillan F., Kirk A., Mutrie N., Matthews L., Robertson K., Saunders D.H. (2014). A systematic review of physical activity and sedentary behavior intervention studies in youth with type 1 diabetes: Study characteristics, intervention design, and efficacy. Pediatric Diabetes.

[41] Roviello V., Gilhen-Baker M., Vicidomini C., Roviello G.N. (2021). Forest-bathing and physical activity as weapons against COVID-19: A review. Environ. Chem. Lett..

[42] Agra G.R.P.D.O., Montenegro M.D.S., Santos M.G.D. (2020). Fatores Associados às Doenças Crônicas Não Transmissiveis na População Brasileira: VIGITEL, 2019.

